# Characterization of *OsPIN2* Mutants Reveal Novel Roles for Reactive Oxygen Species in Modulating Not Only Root Gravitropism but Also Hypoxia Tolerance in Rice Seedlings

**DOI:** 10.3390/plants13040476

**Published:** 2024-02-07

**Authors:** Bowen Hao, Ruihan Zhang, Chengwei Zhang, Na Wen, Yu Xia, Yang Zhao, Qinying Li, Lei Qiao, Wenqiang Li

**Affiliations:** 1 College of Life Sciences, Northwest A&F University, Yangling 712100, China; hbw@nwafu.edu.cn (B.H.); ruihan126@outlook.com (R.Z.); z1259920051@163.com (C.Z.); nave39@163.com (N.W.); xiayu2003@nwafu.edu.cn (Y.X.); zy0508@nwafu.edu.cn (Y.Z.); li15725480698@163.com (Q.L.); leiqiao@nwafu.edu.cn (L.Q.); 2 State Key Laboratory for Crop Stress Resistance and High-Efficiency Production, Northwest A&F University, Yangling 712100, China

**Keywords:** rice, *OsPIN2*, auxin transport, root gravitropism, reactive oxygen species (ROS), hypoxia tolerance

## Abstract

Tolerance to submergence-induced hypoxia is an important agronomic trait especially for crops in lowland and flooding-affected areas. Although rice (*Oryza sativa*) is considered a flood-tolerant crop, only limited cultivars display strong tolerance to prolonged submergence and/or hypoxic stress. Therefore, characterization of hypoxic resistant genes and/or germplasms have important theoretical and practical significance for rice breeding and sustained improvements. Previous investigations have demonstrated that loss-of-function of *OsPIN2*, a gene encoding an auxin efflux transporter, results in the loss of root gravitropism due to disrupted auxin transport in the root tip. In this study, we revealed a novel connection between *OsPIN2* and reactive oxygen species (ROS) in modulating root gravitropism and hypoxia tolerance in rice. It is shown that the *OsPIN2* mutant had decreased accumulation of ROS in root tip, due to the downregulation of glycolate oxidase encoding gene *OsGOX6*, one of the main H_2_O_2_ sources. The morphological defects of root including waved rooting and agravitropism in *OsPIN2* mutant may be rescued partly by exogenous application of H_2_O_2_. The *OsPIN2* mutant exhibited increased resistance to ROS toxicity in roots due to treatment with H_2_O_2_. Furthermore, it is shown that the *OsPIN2* mutant had increased tolerance to hypoxic stress accompanied by lower ROS accumulation in roots, because the hypoxia stress led to over production of ROS in the roots of the wild type but not in that of *OsPIN2* mutant. Accordingly, the anoxic resistance-related gene *SUB1B* showed differential expression in the root of the WT and *OsPIN2* mutant in response to hypoxic conditions. Notably, compared with the wild type, the *OsPIN2* mutant displayed a different pattern of auxin distribution in the root under hypoxia stress. It was shown that hypoxia stress caused a significant increase in auxin distribution in the root tip of the WT but not in that of the *war1* mutant. In summary, these results suggested that *OsPIN2* may play a role in regulating ROS accumulation probably via mediating auxin transport and distribution in the root tip, affecting root gravitropism and hypoxic tolerance in rice seedlings. These findings may contribute to the genetic improvement and identification of potential hypoxic tolerant lines in rice.

## 1. Introduction

In recent years, as climate change leads to more severe weather extremes, submergence and waterlogging events are likely to become even more frequent and severe. Submergence or waterlogging resulting in root-zone hypoxia stress has become one of the major abiotic stresses for plant survival [1,2]. Hypoxia in plants could depress cellular respiration, substance metabolism, and plant growth and development [1,2]. In the lowland and flooding-affected area, hypoxia stress has become the most important adverse environmental factor that causes large loss of crop production [3,4]. Even for rice that adapts to grow in water-logged paddy fields, long time hypoxia stress may cause severe harm to rice production [5,6]. Therefore, exploration of genes or germplasm resistant to hypoxia stress is of great importance for genetic improvement in rice.

Extensive studies have shown that reactive oxygen species (ROS), such as superoxide (O_2•_^−^), hydrogen peroxide (H_2_O_2_) and hydroxyl radicals, play important roles in plant growth, development, and responses to various biotic and abiotic stresses [7,8]. For instance, plants suffering submergence or waterlogging will generally induce root-zone hypoxia and lead to increased ROS accumulation in root cells. Over accumulation of ROS can disturb cellular homeostasis between antioxidants and oxidants that will lead to oxidative stress, trigger membrane lipid peroxidation, and adversely affect membrane functions, and cause leakage of electrolytes, and serious damage of DNA and proteins [9,10]. In this respect, lower ROS accumulation during and following submergence may be associated with increased hypoxic tolerance [11,12]. However, on the other hand, ROS act as signaling molecules to regulate cellular signal transduction and various cellular processes at the physiological level [7]. This is particularly important regarding cell proliferation and differentiation in root growth and development [13]. In Arabidopsis, *UPBEAT1* (*UPB1*) may directly regulate the expression of a set of peroxidases that modulate the balance of ROS between the zones of cell proliferation and the zones of cell elongation where differentiation begins in roots [14]. It is shown that higher levels of ROS can repress *PHB3* expression and consequently activate the expression of AP2/ERF transcriptional factors *ERF109* and *ERF114*, which promote root elongation and differentiation in the elongation and differentiation zones, while reduced ROS levels activate *PHB3* which inhibits *ERF115* to maintain the pluripotency of the quiescent center (QC) [15]. Moreover, in *Arabidopsis thaliana*, H_2_O_2_ treatment promotes root elongation by accelerating cell expansion in the *myb30* mutant [16]. These investigations demonstrated that ROS signals may play extensive roles in regulating root growth and development as well as responses to various environmental stresses.

Phytohormones are the most important signaling molecules in plant cells that regulate a large range of developmental and physiological processes. Increasing evidence has shown a close connection between ROS and phytohormones in regulating many aspects of plant growth and development [17,18,19]. The roles for ROS–hormone crosstalk in regulating root cell proliferation and differentiation and related processes have been demonstrated in many previous investigations [13,16,20,21]. For instance, accumulated ethylene enhances ROS production and subsequently promotes rice root elongation under flooding conditions [22]. It is revealed that H_2_O_2_ generation is involved in auxin-mediated lateral root formation [23], and exogenous ROS such as H_2_O_2_ treatment may mimic lateral root induction mediated by auxin [24]. Moreover, it is proposed that auxin may induce ROS production through the modulation of the NAD(P)H oxidase RBOHD activity [25]. These investigations suggest that ROS–auxin interactions may play an important role in root cell proliferation and differentiation and root formation.

For higher plants, how the root senses gravity and directs its orientation, the so-called gravitropism, is a fundamental question in plant biology. Although extensive studies have shown the crucial role for polar auxin transport in regulating root gravitropism, ROS signals are also involved in gravitropic signaling [26,27,28]. It is revealed that gravistimulation or unilateral application of auxin to vertical roots leads to a transient increase in intracellular ROS concentration in the root endodermis, while unilateral application of ROS to vertical roots pretreated with auxin transport inhibitor may also lead to gravitropic bending [29]. Furthermore, scavenging of ROS by antioxidants may inhibit root gravitropism, indicating the role of ROS in regulating root gravitropism [29]. These investigations suggest that the crosstalk between ROS and auxin may modulate root gravitropism and related physiological processes. Several previous studies have shown that the loss-of-function of rice *OsPIN2*, a gene encoding an auxin efflux transporter, led to the loss of root gravitropism due to disrupted auxin transport and distribution in the root cap [30,31,32]. However, it remains unknown if additional signals were involved in *OsPIN2*-mediated auxin transport in regulating root gravitropism. Here, we report a novel connection between *OsPIN2* and ROS signals in modulating not only root gravitropism but also hypoxic tolerance in rice seedlings. The findings will contribute to a novel understanding of the molecular mechanism for root gravitropism and genetic improvement of hypoxic tolerance in rice.

## 2. Results

### 2.1. OsPIN2 Mutant Showed Lower ROS Accumulation in Root Tip

**Figure 1:**
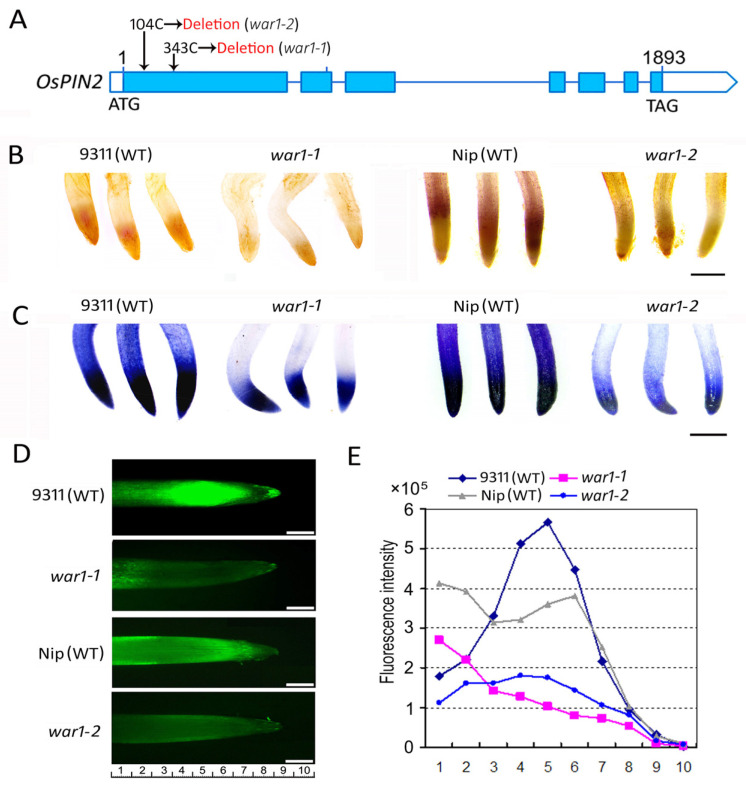
The *OsPIN2* mutants *war1-1* and *war1-2* showed lower ROS levels in the toot tips. (**A**) Schematic diagram of *war1-1* and *war1-2* mutation site in the *OsPIN2* locus. Single base-pair deletions (104C deletion in *war1-2* and 343C deletion in *war1-1*) were shown. (**B**,**C**) 3,3′-diaminobenzidine (DAB) and nitrotetrazolium blue chloride (NBT) histochemical staining were used to detect the levels of ROS hydrogen peroxide (**B**) and superoxide anion (**C**) in the root tips of WT (cv. 9311 and cv. Nip) and *OsPIN2* mutants. (**D**) Detection of ROS (H_2_O_2_ and O_2•_^−^) accumulation by fluorescent staining with H_2_DAF-DA in the root tip of WT (cv. 9311 and cv. Nip) and *OsPIN2* mutants. (**E**) ROS fluorescence intensity at the corresponding position of the root tip. The numbers underneath indicate the position from the root tips corresponding to that in (**D**). Scale bars = 1 mm (**B**,**C**), and 500 μm (**D**).

The *OsPIN2* mutants, *war1-1* and *war1-2*, were derived from *Oryza sativa* L. *indica* cv. 9311 and *Oryza sativa* L. *japonica* cv. Nipponbare (Nip), respectively. The *war1-1* and *war1-2* mutant were also indicated as *war1* and *war1-cr#3* in the previous study [30,31,32]. As is shown in Figure 1A, both *war1-1* and *war1-2* contain single base-pair deletions in the coding region of *OsPIN2*, representing the loss-of-function alleles in the *OsPIN2* locus. Histochemical analysis was performed on the WT (cv. 9311 and cv. Nip) and *OsPIN2* mutants using 3,3′-diaminobenzidine (DAB) and nitrotetrazolium blue chloride (NBT) staining to determine ROS (hydrogen peroxide and superoxide anion) production in the root tip. The results from DAB histochemical staining showed that the root tips of two WTs (cv. 9311 and cv. Nip) have darker staining, but the *OsPIN2* mutants *war1-1* and *war1-2* have shallower staining (Figure 1B), indicating that the *OsPIN2* mutants have less hydrogen peroxide (H_2_O_2_) accumulation in the root tip. The results from NBT histochemical staining also showed that *war1-1* and *war1-2* were less stained in the root tip (Figure 1C), indicating that less accumulation of superoxide anion (O_2•_^−^) in the root tip of *OsPIN2* mutants. 2′,7′-dichlorodihydrofluorescein diacetate (H_2_DAF-DA) as a fluorescence probe may detect intracellular ROS (H_2_O_2_ and O_2•_^−^) and has been routinely used to quantify ROS in plant cells. We therefore used a H_2_DAF-DA fluorescence probe to measure the ROS levels in the WT and the mutants. The results showed that both *war1-1* and *war1-2* have weaker intensity of fluorescence signals in the root tip as compared with WT (Figure 1D,E), further indicating less accumulation of ROS in the *OsPIN2* mutants.

### 2.2. Expressions of ROS-Generating Genes in the Root of OsPIN2 Mutant

**Figure 2:**
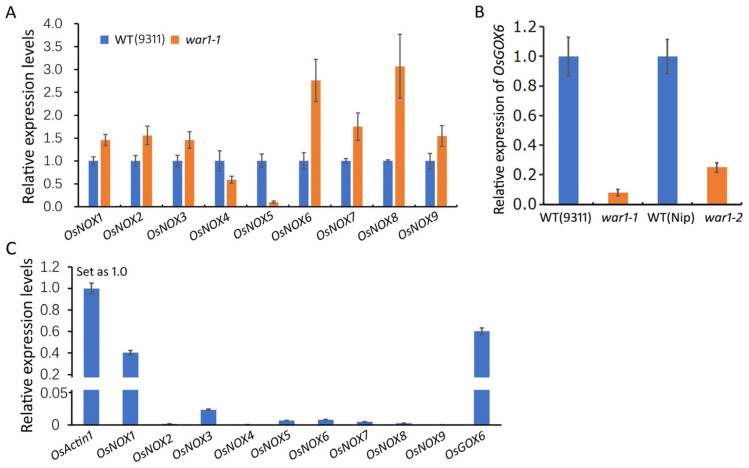
Expression levels of ROS-generating genes *OsNOX1~9* and *OsGOX6* in the root of WT and *OsPIN2* mutants. (**A**) Relative expression of *OsNOX1~9* in the root of WT (cv. 9311) and the *war1-1* mutant. (**B**) Relative expression of *OsGOX6* in the WT (cv. 9311 and cv. Nip) and the *OsPIN2* mutants (*war1-1* and *war1-2*). The expression levels in (**A**,**B**) were analyzed by the 2^−ΔΔCt^ method. (**C**) Relative expression levels of *OsNOX1~9* and *OsGOX6* in the root of WT (cv. 9311) as compared with internal reference gene *OsActin1*. The expression of *OsActin1* was set as 1. The expression levels of *OsNOX1~9* and *OsGOX6* were analyzed according to the 2^−ΔCt^ method.

Previous study by Wang et al. [33] had reported that the rice genome encodes nine NADPH oxidase genes (*OsNOX1-*9), also known as respiratory burst oxidase homologues (RBOH) genes, which are responsible for ROS generation in the plasma membrane and extracellular space. To investigate if the NADPH oxidase genes are responsible for the decrease in ROS in the *OsPIN2* mutants, we analyzed the expressions of *OsNOX1~9* genes in the root tips of the WT (cv. 9311) and *war1-1* by RT-qPCR. It is revealed that the expressions of *OsNOX1*, *OsNOX2*, *OsNOX3*, *OsNOX7* and *OsNOX9* were slightly upregulated while *OsNOX4* was slightly downregulated in the root of the *war1-1* mutant as compared with that of the WT (Figure 2A). The expressions of *OsNOX6* and *OsNOX8* were more than 2-folds upregulation in the root tip of the *war1* mutant, while *OsNOX5* showed more than 6-folds downregulation in the *war1-1* mutant (Figure 2A). In fact, ROS are not only produced in the cell membrane and extracellular space, but also are generated continuously in the mitochondria and chloroplasts. According to our previous RNA-seq data [33], it showed that *OsGOX6 (LOC_Os08g09860)*, a gene encoding glycolate oxidase or called hydroxyacid oxidase that catalyzes the oxidation in vivo of glycolic acid to glyoxylic acid and hydrogen peroxide, was significantly downregulated in the root of the *war1-1* mutant. The RNA-seq data showed that the mRNA levels of *OsGOX6* were 87.72, 100.03, and 90.57 in three independent biological replicates of WT (cv. 9311), but were 0, 0, and 0.06 in the three biological replicates of the *war1-1* mutant. We further performed RT-qPCR to analyze the expression of *OsGOX6* in the root of the WT (cv. 9311 and cv. Nip) and the two mutant lines. It was shown that the expression of *OsGOX6* was down-regulated by 13-folds in the *war1-1* mutant as compared with that in cv. 9311 (Figure 2B). Compared with that in cv. Nip, the expression of *OsGOX6,* was decreased by 4-folds in the root of the *war1-2* mutant (Figure 2B). To explore which of these ROS-generating genes make more contribution to produce ROS in roots, we further compared their relative expression levels to reference gene *OsActin1* in the root of the WT (Figure 2C). It was shown that the *OsNOX1*, *OsNOX3* and *OsGOX6* had relatively high expression levels in rice root (Figure 2B), indicating *OsNOX1*, *OsNOX3* and *OsGOX6* may have more contribution to produce ROS in rice root. Since *OsNOX1* and *OsNOX3* were slightly upregulated in the root of the *war1-1* mutant and *OsGOX6* was 13-folds down-regulated in the root of the *war1-1* mutant, the results suggest that the downregulated expressions of *OsGOX6* in *war1-1* and *war1-2* were possibly responsible for the decreased production of ROS in the root tip of *OsPIN2* mutant lines.

### 2.3. Application of Hydrogen Peroxide Induces Root Gravitropic Curvature in OsPIN2 Mutant

**Figure 3:**
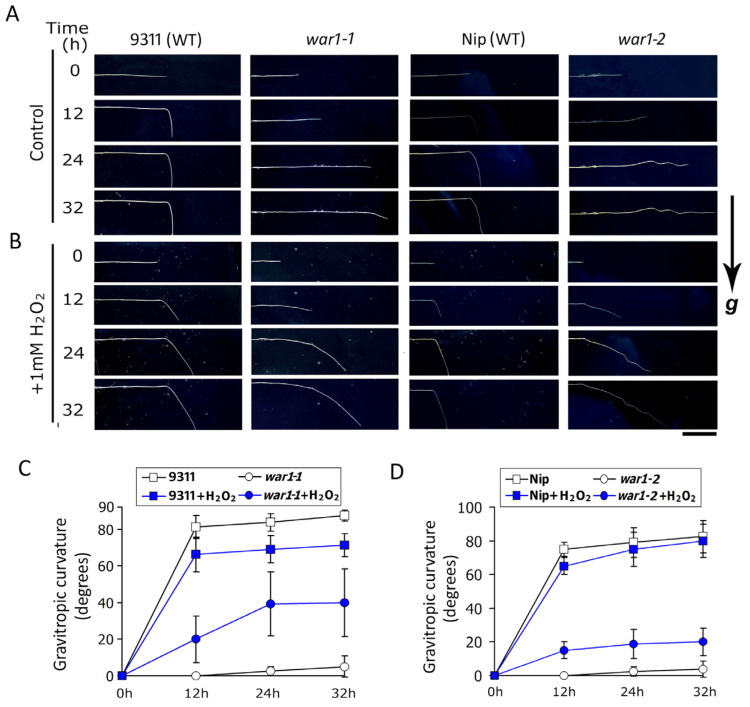
The *OsPIN2* mutants, *war1-1* and *war1-2*, showed gravitropic curvature in root tip by application of hydrogen peroxide. (**A**,**B**) The roots of *war1-1*, *war1-2* and their respective WT (cv. 9311 and cv. Nip) were embedded horizontally in vertical agar plates containing no H_2_O_2_ (**A**) or 1 mM H_2_O_2_ (**B**). Root images were captured at 0, 12, 24, and 32 h after embedding into the vertical plate. The direction of gravity is indicated by “g”. Scale bar = 1 cm. (**C**,**D**) Degrees of gravitropic curvature of root tip in cv. 9311 and *war1-1* (**C**), and cv. Nip and *war1-2* (**D**) at 0, 12, 24, and 32 h after embedding. Values are means ± SD (*n* = 6).

Previous investigations have shown that the *OsPIN2* mutants lack root gravitropism due to disrupted auxin transport in the root tip [30,31,32]. In this study, the results that *OsPIN2* mutants had less ROS accumulation in the root tip promoted us to investigate if exogenous application of hydrogen peroxide could rescue the gravitropic defects in *war1*. It is shown that the root tips of WT (cv. 9311 and cv. Nip) exhibited gravitropic curvature, while *war1-1 and war1-2* had no gravitropic curvature at non-application with H_2_O_2_ (Figure 3A). However, applications of H_2_O_2_ induced gravitropic curvature of the root tip in *war1-1 and war1-2* (Figure 3B–D). This result indicated that application of hydrogen peroxide may induce root gravitropic curvature in *OsPIN2* mutants.

### 2.4. OsPIN2 Mutant Showed Increased Resistance to ROS Toxicity in Roots

**Figure 4:**
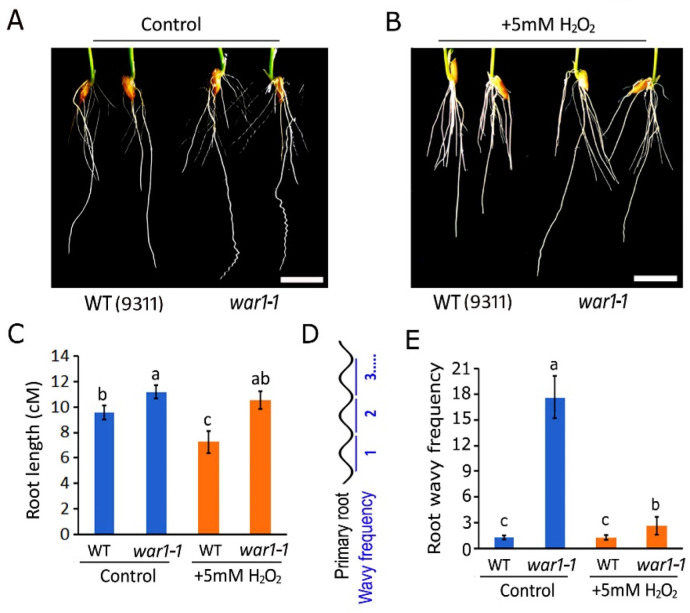
The *OsPIN2* mutant showed increased resistance to ROS hydrogen peroxide toxicity. (**A**,**B**) Germinated seeds of WT (cv. 9311) and *war1-1* were cultured in hydroponic medium supplemented with no H_2_O_2_ (**A**) or 5 mM H_2_O_2_ (**B**) for 8 days. Scale bars = 1 cm (**A**,**B**). (**C**) Root length for WT (cv. 9311) and *war1-1* cultured for 8 days in hydroponic medium supplemented with no H_2_O_2_ (control) or 5 mM H_2_O_2_. (**D**) Schematic diagram of wavy frequency for each primary root. (**E**) Wavy frequency for primary root in WT (cv. 9311) and *war1-1* cultured in hydroponic medium supplemented with no H_2_O_2_ (control) or 5 mM H_2_O_2_. Values are means ± SD (*n* = 12). The letters above the bars indicate significant differences (*p* < 0.05) as determined by one-way ANVOA followed by Tukey’s test.

The findings that *OsPIN2* mutants have less ROS accumulation in the root tip and applications of H_2_O_2_ may induce gravitropic curvature in the root tip promoted us to further investigate if *OsPIN2* mutants are more resistant to an exogenous application of a higher concentration of hydrogen peroxide. It showed that the application of 5 mM H_2_O_2_ in hydroponic medium may cause stronger repression of root elongation in WT than *war1-1*, as compared with that of the control (Figure 4A,B). The root length was significantly decreased in the WT but there was no significant change in *war1-1* by application of H_2_O_2_ in the hydroponic medium (Figure 4C). The result indicated that *OsPIN2* mutant had increased resistance to ROS toxicity. Moreover, we found that the application of H_2_O_2_ markedly represses the wavy rooting phenotype in *war1-1*, as compared with that of control (Figure 4A,B). The wavy frequency for the primary root was significantly decreased in *war1-1* by application of H_2_O_2_ in a hydroponic medium (Figure 4D,E).

### 2.5. OsPIN2 Mutant Showed Lower ROS Accumulation and Stronger Tolerance under Hypoxic Stress

**Figure 5:**
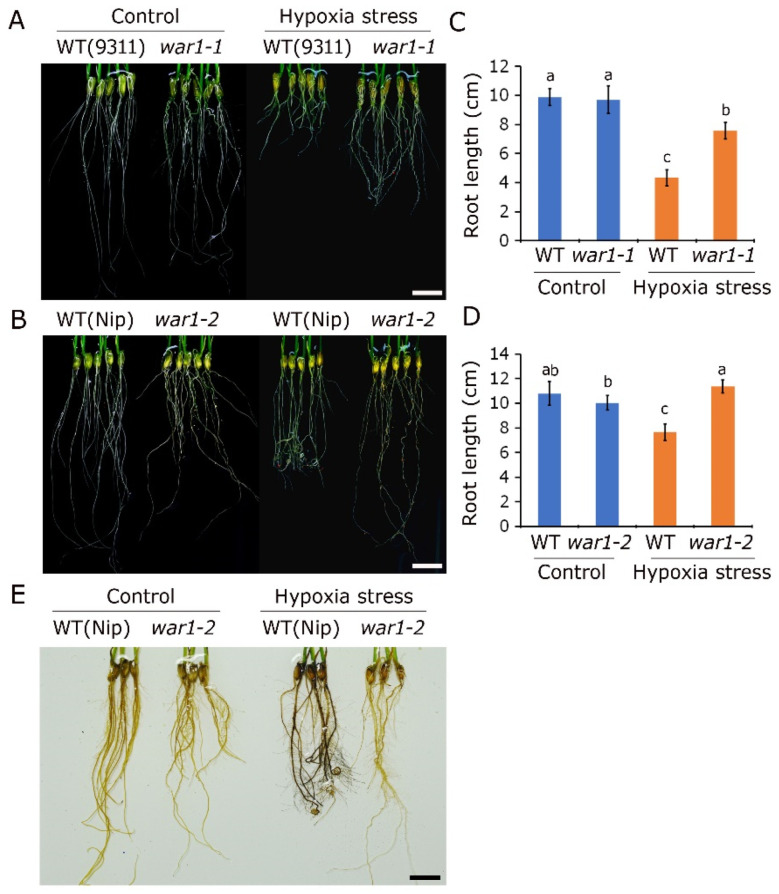
The *OsPIN2* mutant showed enhanced tolerance and decreased ROS accumulation under hypoxic stress. (**A**,**B**) The phenotypes of roots of WT (cv. 9311 and cv. Nip) and *OsPIN2* mutants (*war1-1* and *war1-2*) after non-stress (control) or hypoxic stress. (**C**,**D**) The root length of WT (cv. 9311 and cv. Nip) and *OsPIN2* mutants (*war1-1* and *war1-2*) after non-stress (control) or hypoxic stress. Values are means ± SD (*n* = 12). The letters above the bars indicate significant differences (*p* < 0.05) as determined by one-way ANVOA followed by Tukey’s test. (**E**) Detection of H_2_O_2_ accumulation by DAB staining in the root of Nip and *OsPIN2* mutant *war1-2* under non-stress (control) or hypoxic stress conditions. Scale bars = 2 cm (**A**,**B**,**E**).

Because rice plants have higher background tolerance to hypoxia stress than upland crops, it is difficult to kill the plants by hypoxia stress under sufficient nutritional conditions. We therefore performed a hypoxia experiment to test the effects of hypoxia stress on root growth. It is shown that the root growth was significantly inhibited in the WT (cv. 9311 and cv. Nip) by 10 days of hypoxia stress with 0.1% agar solution (Figure 5A,B). It is interesting that hypoxia stress results in root tip curling in cv. Nip, but not in the *war1-2* mutant (Figure 5B). Compared with wild type 9311 and Nip, hypoxia stress had a smaller inhibition on the root growth in *war1-1* and *war1-2* (Figure 5A,B). As is shown in Figure 5C, the hypoxia stress caused a 56.1% decrease in root length in 9311 but caused only a 22.3% decrease in root length in *war1-1*. It is surprising that the hypoxia stress did not cause a decrease in root length in *war1-2* (Figure 5D). As is shown in Figure 5D, the hypoxia stress caused a 29.9% decrease in root length in Nip but caused a 13.3% increase in root length in *war1-2*. ROS detection by DAB staining was further performed in roots of Nip and *war1-2*. It is revealed that hypoxia stress led to increased accumulation of ROS in the roots of Nip but caused no significant increase in ROS accumulation in the roots of *war1-2* (Figure 5E), indicating that the *OsPIN2* mutation inhibited the production of ROS in roots and led to enhanced resistance to hypoxia stress. These results suggested that *OsPIN2* mutants exhibiting stronger hypoxia tolerance were possibly associated with less ROS accumulation under hypoxic stress, because high levels of ROS accumulation are lethal to root cells.

### 2.6. Differential Expression of Anoxic Resistance-Related Gene SUB1B in the Root of WT and OsPIN2 Mutant under Hypoxic Stress

**Figure 6:**
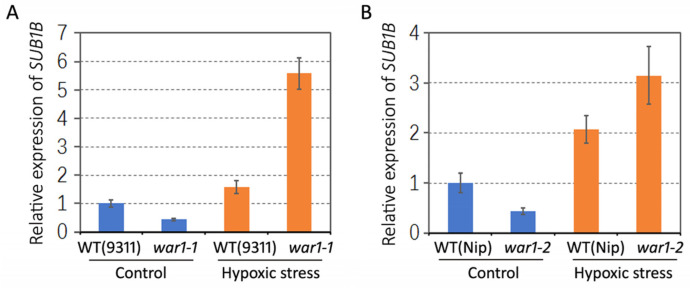
Relative expressions of anoxic resistance-related gene *SUB1B* in the root of WT and *OsPIN2* mutants under non-stress (control) or hypoxic stress conditions. (**A**) Expression levels of *SUB1B* in WT (cv. 9311) and *war1-1* mutant. (**B**) Expression levels of *SUB1B* in WT (cv. Nip) and *war1-2* mutant.

According to our previous RNA-seq data [34], *SUB1B* (LOC_Os09g11460), an anoxic or submergence resistance-related gene, was significantly downregulated in the *war1* mutant under non stressed conditions. It is shown that the mRNA levels of *SUB1B* were 12.02, 11.2, and 9.6 in the three independent biological replicates of WT, but were 5.31, 3.8, and 4.31 in the three biological replicates of the *war1-1* mutant. In this study, we further investigate the gene expression of *SUB1B* in the root of the WT (cv.9311 and cv. Nip) and *OsPIN2* mutants (*war1-1* and *war1-2*) under non-stress or hypoxia stress conditions. The results from RT-qPCR showed that the *SUB1B* gene was downregulated in the root of *war1-1* and *war1-2* under non-stressed condition, as compared with that of the WT (Figure 6A,B). However, the hypoxia stress caused upregulations of *SUB1B* expressions in the WT (cv. 9311 and cv. Nip) but caused stronger upregulations of *SUB1B* in the root of *war1-1* and *war1-2* mutants (Figure 6A,B). The result suggested that loss-of-function of *OsPIN2* may induce expression of *SUB1B* in response to hypoxia stress.

### 2.7. OsPIN2 Mutant Showed Reduced Auxin Distribution in the Root Tip under Hypoxia Stress

**Figure 7:**
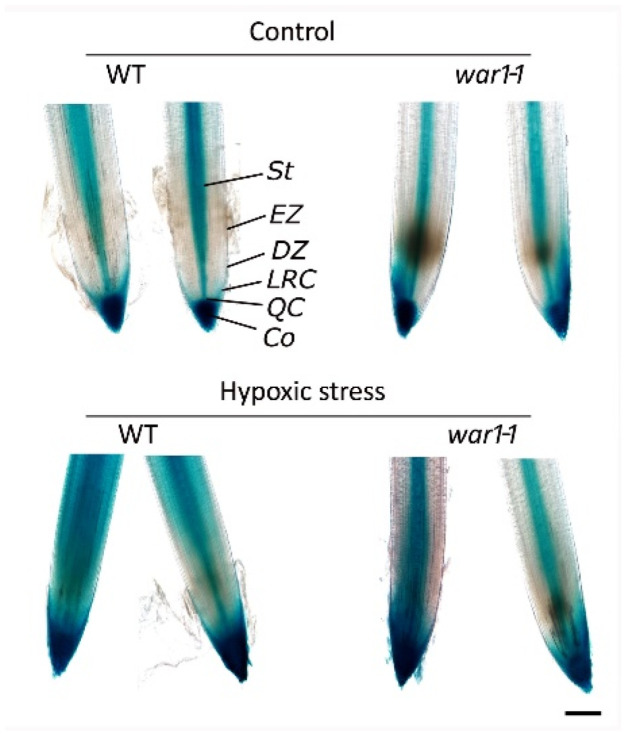
Detection of auxin distribution in root tip by *DR5 promoter-GUS* experiment. *GUS* reporter gene under the control of auxin-inducible *DR5 promoter* was expressed in cv. 9311 (WT) and *war1-1*. GUS staining was performed with root tip from primary root of 10-day-old seedling. *St*, stele; *EZ*, elongation zone; *DZ*, division zone; *Co*, columella; *LRC*, lateral root cap; *QC*, quiescent center. Scale bars = 200 μm.

Since the roots of *OsPIN2* mutant had stronger hypoxia tolerance and less ROS accumulation under hypoxic conditions, we speculated that these changes might be associated with disrupted auxin transport in the root tip. Therefore, we performed a *DR5 promoter-GUS* experiment to investigate auxin distribution in the root tip in response to hypoxia stress. It showed that WT and *war1-1* mutant have different patterns of auxin distribution and accumulation in the root tip in response to hypoxic stress (Figure 7). Under non-stress conditions, auxin IAA as represented by GUS signals was mainly accumulated in the whole root cap, QC, and central vascular tissue in WT (Figure 7), but was confined to the stele, QC, columella and one side of the lateral root cap in *war1-1* (Figure 7). Under hypoxic stress conditions, the distribution of auxin was altered in both the WT and *war1-1* as compared with that in non-stress condition (control). It showed that hypoxia stress induced auxin distribution not only in the stele but also diffused distribution throughout the elongation zone in the root tip in the WT (Figure 7). However, auxin distribution was still located mainly in the root stele in the *war1-1* mutant under hypoxia stress (Figure 7). Compared with one side of auxin distribution in the *war1-1* root cap under non-stressed conditions (control), the hypoxic stress induced auxin distribution at two sides of the root cap in the *war1-1* (Figure 7). Moreover, there has been a trend that auxin was diffused from the root cap to the meristem or division zone (Figure 7). Overall, it is noteworthy that hypoxic stress caused a significant increase in auxin distribution and accumulation in the root tip in the WT but caused a slight increase in auxin accumulation in the root tip of the *war1-1* (Figure 7).

## 3. Discussion

Extensive studies have demonstrated that PIN-FORMED (PIN) auxin efflux carriers, especially PIN2, are required for root gravitropism in plants [30,31,32,35,36], indicating the importance for auxin transport in regulating gravitropism [27]. Besides the hormone auxin, additional signal molecules such as Ca^2+^ [37,38], inositol 1,4,5-trisphosphate [39] and nitric oxide [40] are also necessary for gravitropism signal transduction and responsive processes. Furthermore, there is evidence that reactive oxygen species (ROS) are implicated in regulating root gravitropic perception [28]. ROS may accumulate on the lower side of roots in an auxin-dependent manner, where they have been shown to modulate the gravitropic curvature [29]. Since application of hydrogen peroxide may induce curvature even in roots treated with auxin transport inhibitor NPA, it is believed that ROS functions downstream of auxin signals [41]. Our previous study indicates that the *OsPIN2* mutant, *war1*, produces a waved rooting phenotype due to the loss of root gravitropism [32]. In this study, application of hydrogen peroxide may rescue the waved rooting phenotype in the *OsPIN2* mutant (Figure 4). The root tip of the *OsPIN2* mutant showed no gravitropic curvature, but application of hydrogen peroxide may induce gravitropic curvature in the mutant (Figure 3), indicating that ROS may induce a root gravitropic response in *OsPIN2* mutant. This result may suggest that ROS signals regulating root gravitropism are independent of *OsPIN2* function. This conclusion was consistent with the previous hypothesis proposed by Joo et al. (2005) [41] that ROS signals act as a downstream component of the auxin-mediated root geotropism signaling pathway.

Many investigations have shown that ROS are essential for numerous developmental and physiological processes throughout the plant life cycle [7,8]. the importance of ROS as growth regulators to modulate root developmental processes such as meristem maintenance, root elongation, lateral root and root hair formation, and endodermis and vascular differentiation has been indicated [13,42]. In fact, these processes generally involve a complex interplay between steady-state levels of ROS and phytohormone signals [13]. In this study, we focused on the connection between ROS and auxin actions since they have extensive interactions in regulating root growth and development. It is shown that localized auxin accumulation may increase ROS in most cell types [25]. In Arabidopsis, RBOH-mediated ROS production may facilitate lateral root emergence, suggesting the function of ROS as important signals during auxin-regulated lateral root formation [43]. Investigations on tomato (*Solanum lycopersicum*) further revealed that auxin regulates the level of H_2_O_2_ in the root tip and an increase in auxin level may trigger accumulation of H_2_O_2_ leading to inhibition of root cell elongation and root growth [44]. It has been revealed that the loss of *OsPIN2* function altered auxin transport and distribution in the root, thus leading to abnormal root system architecture, root elongation growth and lateral root formation patterns [30,31,32]. In this study, our data indicated that the loss of *OsPIN2* function led to reduced H_2_O_2_ accumulation in the root tip (Figure 1), and the root defects in the *OsPIN2* mutant may be rescued partly by application of H_2_O_2_, suggesting that *OsPIN2*-mediated auxin transport and distribution regulate root growth and development probably by modulating ROS homeostasis in the root tip. In plant cells, besides production by NADPH oxidase (or called RBOH), an alternative ROS-producing pathway can be mediated by glycolate oxidase (GOX) [45], because GOX catalyzes glycolate or its derivatives into glyoxylate and H_2_O_2_ during photorespiration [46]. It is confirmed in *Nicotiana benthamiana* leaves that transgenic plants’ overexpression of GOX induces ROS accumulation [47]. In rice, it has been demonstrated that GOX isoforms are localized to the peroxisome [48]. The physical association–dissociation of GOX and catalase, in response to environmental stress, seems to serve as a specific mechanism to modulate H_2_O_2_ levels in rice [45]. In fact, the genes encoding GOX are not only present in plants and green algae, but are also widely found in animals and bacteria [49,50]. It is considered that plant and animal GOXs have a common eukaryotic ancestor and animal cells also possess GOX activities which produce glyoxylate used for the peroxisomal synthesis of glycine [49,50,51], indicating that GOX may catalyze glycolate into glyoxylate and by-product H_2_O_2_ in non-photorespiratory pathways. In humans, glycolate oxidase is a potential drug target for treatment of primary hyperoxaluria, a genetic disorder where overproduction of oxalate results in the formation of kidney stones [52,53]. A previous investigation suggested that the *Ricinus communis* genome encodes a single GOX with different functions in photosynthetic and heterotrophic organs [54]. Schmitz et al. [54] reporting a high level of glyoxylate in *Ricinus communis* roots have hypothesized that in heterotrophic tissues such as the root, the GOX is involved in the production of serine (from glyoxylate) to feed the folate pathway. In Arabidopsis, however, it was shown that GOX3, a glycolate oxidase homolog of yeast l-lactate cytochrome *c* oxidoreductase, is predominantly present in roots and may catalyze l-lactate oxidation [55]. In fact, more convincing evidence came from the study of rice that *OsGLO4*, a glycolate oxidase gene, is involved in the formation of iron plaque on the surface of roots by mediating GOX activity and H_2_O_2_ production under alternative wetting and drying condition [56]. In the present study, the GOX encoding gene *OsGOX6* was greatly downregulated in the root of *OsPIN2* mutants (Figure 2), suggesting that glycolate metabolism in roots was depressed due to the lack of *OsPIN2* function. As indicated previously, GOX can also catalyze glycolate into glyoxylate and H_2_O_2_ during non-photorespiratory pathways; the downregulated expression of *OsGOX6* could be responsible for the lower levels of H_2_O_2_ in the root of the *OsPIN2* mutant. Since *OsPIN2* encodes an auxin efflux transporter and its absence may disrupt the polar auxin transport in the root tip and lead to abnormal auxin levels in the root cap [30,31,32]; our data may have indicated an interesting connection between *OsPIN2*-modulated auxin transport and *OsGOX6*-mediated H_2_O_2_ production in rice root. 

It is widely believed that high levels of ROS can be lethal for plant cell integrity [15], although cell death in response to biotic or abiotic stresses is often mediated by plant hormones. At lower concentrations, ROS may function in signaling pathways that regulate plant development in response to physiological and environmental stresses [57]. In this study, the *OsPIN2* mutant showed less ROS accumulation in the root tip under non-stressed conditions. Hypoxia stress caused no significant increase in the ROS level in the *OsPIN2* mutant root but overproduction of ROS in the WT (Figure 5). Since high levels of ROS may be lethal for the plant cell integrity, we draw a conclusion that the *OsPIN2* mutant had stronger root activity under hypoxia stress due to less ROS accumulation in the root. Consistent with this conclusion, the hypoxia stress caused less inhibition of root growth in the *OsPIN2* mutant but resulted in significant inhibition of root growth in the WT, suggesting that loss of *OsPIN2* function led to enhanced resistance to hypoxia stress, owing to lower ROS accumulation in roots. We suggested that root growth inhibition in the WT was due to ROS-induced cell death in root cells. Previous investigations have suggested that hydrogen peroxide (H_2_O_2_) is required for ethylene-induced epidermal death and promotes the aerenchyma development in rice stems [58,59]. Furthermore, it is revealed that exposure of plant cells to different concentrations of H_2_O_2_ may induce different cell death pathways in a dose-dependent manner [60,61]. In the present study, the root elongation was significantly decreased in the WT but was not significantly decreased in the mutant by application of H_2_O_2_, suggesting that the *OsPIN2* mutant was more resistant to ROS toxicity. This was consistent with the fact that the *OsPIN2* mutant accumulated lower levels of ROS in the roots. These results indicated that loss-of-function of *OsPIN2* led to a reduction in ROS accumulation and increase in root resistance to ROS toxicity.

Tolerance to flooding-induced hypoxia is an important agronomic trait especially for crops in lowland and flooding-affected areas. Although rice is considered a flood-tolerant crop, only limited cultivars display tolerance to prolonged submergence, which is largely attributed to the presence of the *SUB1A*, *a* gene encoding the AP2/ERF transcriptional factor [6,62]. However, for most rice cultivars, such as cv. Nipponbare (*O. sativa* L. ssp. *japonica*) and cv. 9311 (*O. sativa* L. ssp. *indica*), the *SUB1A* allele is absent in the genome. Alternatively, these cultivars possess two alleles of the *SUB1 orthologue*, *SUB1B* (LOC_Os09g11460, https://www.ricedata.cn/gene/list/365.htm, accessed on 1 November 2023) and *SUB1C* (LOC_Os09g11480, https://www.ricedata.cn/gene/list/362.htm, accessed on 1 November 2023), in the genome. In this study, it is shown that *SUB1B* expression was significantly decreased in the roots of the *OsPIN2* mutant under non-stressed conditions, indicating that the loss of the *OsPIN2* function led to repression of *SUB1B* expression. Considering that the *SUB1*-meidated resistant pathway is related to anaerobic respiration, plant hormone response, and the antioxidant system [5], it seems that enhanced hypoxic tolerance in the *OsPIN2* mutant was associated with strong induction of *SUB1B* expression under hypoxia stress. Previous investigations had shown that a set of AP2/ERF transcriptional factors are ROS responsive in Arabidopsis root. For instance, *ERF109*, *ERF114* and *ERF115* were highly upregulated in response to ROS accumulations, but the AP2/ERF transcriptional factors *PLT1* and *PLT2* were downregulated in their expression in response to ROS accumulation [15]. However, the AP2/ERF transcriptional factor *SUB1B* exhibited opposite patterns of expression between the WT and *OsPIN2* mutant, indicating that additional factors instead of ROS might affect *SUB1B* expression under hypoxic stress.

It is shown that root tips are extremely sensitive to flooding stress and often die after a few hours of exposure to hypoxic conditions, compromising root survival following the re-establishment of normoxic oxygen levels [63]. For instance, maize root tips exposed to 4% oxygen for 24 h undergo massive cellular death, preceded by the accumulation of NO and ROS [64]. Some investigations suggested that root survival to hypoxic stress is dependent upon maintaining the function of the apical root meristem quiescent center (QC). This process is governed by PIN-mediated basipetal flow of auxin leading to the formation of an auxin maximum that is required for the establishment of a highly oxidized environment specifying the QC niche [65]. However, perturbations in auxin flow and/or distribution along root profile during hypoxic stress may shift the redox state of the QC towards a more reduced environment leading to the activation of the QC, degradation of the meristem, and root abortion [65]. The previous investigation indicated that the loss of *OsPIN2* function resulted in abnormal auxin flow or distribution in root cap and QC under non-stressed conditions [30,31,32]. In this study, it was shown that hypoxia stress resulted in higher accumulation of auxin in the root cap and elongation zone in WT but led to less accumulation of auxin in the tip (especially the elongation zone) in the *OsPIN2* mutant. It seems that hypoxia-induced auxin accumulation was associated with ROS accumulation due to the lack of *OsPIN2*. According to our data, hypoxic stress induced higher accumulation for both ROS and auxin in the WT, while led to less accumulation for ROS and auxin in the *OsPIN2* mutant. These results indicated a connection between auxin distribution and ROS accumulation in response to root hypoxia. We suggested that the loss-of-function of *OsPIN2* resulted in decreased auxin transport and accumulation in the root tip, leading to less accumulation of ROS in the root tip, followed by enhanced hypoxia tolerance.

## 4. Materials and Methods

### 4.1. Plant Materials and Growth Conditions

The *OsPIN2* mutants, *war1-1* derived from the rice cv. 9311 (*Oryza sativa* L. *indica*) by ^60^Co γ-ray mutagenesis and *war1-2* (also indicated as *war1-cr#3* previously) derived from the rice cv. Nipponbare (Nip, *Oryza sativa* L. *japonica*) by CRISPR/Cas9-mediated mutagenesis of *OsPIN2*, were characterized as root gravitropic mutants previously [32]. To observe the root phenotype, germinated seeds of wild type (cv. 9311 and cv. Nip) and *OsPIN2* mutants (*war1-1* and *war1-2*) were planted in Hoagland‘s hydroponic solution and supplemented with 0.5 mM MES monohydrate (pH 6.5) as described previously [32]. To investigate the effects of exogenous application of H_2_O_2_ on root growth, germinated seeds were transferred to hydroponic Hoagland’s medium supplemented with 5 mM H_2_O_2_. The hydroponic system was kept in a greenhouse with 12 h light (28 °C) and 12 h dark (25 °C) photoperiod at 180 µmol m^−2^ s^−1^ photon density and 70–80% humidity. The hydroponic culture was refreshed every three days. To examine the effects of exogenous application of H_2_O_2_ on root gravitropism, germinated seed of roots were placed horizontally in MS agar plates that were supplemented with or without 1 mM H_2_O_2_. The plates were then positioned vertically, and the downward bending angle of the root tip was measured at various time points.

### 4.2. Hypoxic Stress Treatment

The hypoxic stress treatment was performed as described by Li et al. [34] with minor modification. For aerobic hydroponic culture (control), oxygen was supplied through an oxygen pump for 6 h daily, and the concentration of dissolved oxygen in the culture medium was maintained above 6 ppm. For hypoxic stress in hydroponic culture, 0.1% agar was added into the Hoagland’s culture medium to create a viscous deoxygenated environment, causing the dissolved oxygen content to decrease to about 0.5–1.0 ppm on the fourth day of culturing. Germinated seeds of wild type and *OsPIN2* mutant were planted on aerobic hydroponic culture and hypoxic culture medium in a greenhouse with 12 h light (28 °C) and 12 h dark (25 °C) photoperiod at 180 µmol m^−2^ s^−1^ photon density and 70–80% humidity.

### 4.3. Detection of ROS Level in Roots

The detection of hydrogen peroxide (H_2_O_2_) and superoxide anion (O_2•_^−^) in roots was performed using 3,3′-diaminobenzidine (DAB) and nitrotetrazolium blue chloride (NBT) histochemical staining, respectively, as described by Xu et al. [66]. Briefly, root tips were immersed directly into 0.5 mg/mL DAB solution (prepared in distilled water, pH 3.8) for 3 h at 25 °C, and the production of hydrogen peroxide was monitored by observing the development of a brown stain in the root tip. For detection of superoxide anion, root tips were immersed directly in a reaction medium containing 0.25 Mm NBT in 50 Mm K-phosphate buffer (Ph 7.8) for 1 h at 25 °C. Superoxide anion was detected by the formation of blue formazan. The total level of hydrogen peroxide and superoxide anion was further measured by fluorescence microscopy after labeling with the cell-permeable fluorogenic probe, 2′,7′-dichlorodihydrofluorescein diacetate (H_2_DCF-DA), as described by Wang et al. (2016) [67] with minor modifications. Briefly, root tips were immersed in 1 Ml of 10 Mm MES-KCl buffer (Ph 6.1), and then 1 µL of 10 Mm H_2_DCF-DA solution was added. After incubating for 8 min in the dark, the roots were washed 4 times with deionized water, and the fluorescence from the H_2_DCF-DA was recorded using a Leica DM5000B fluorescence microscope (Leica, Wetzlar, Germany) with a GFP filter.

### 4.4. RNA Extraction and RT-qPCR Analysis

Total RNA was extracted from 0.5 cm long root tips using the RNeasy Plant Mini Kit (Qiagen 74904, Qiagen Inc., Hilden, Germany) according to the manufacturer’s instructions. First-strand cDNA was synthesized using the M-MLV first strand kit (Invitrogen, Carlsbad, CA, USA) by reverse transcription. Real-time RT-qPCR was performed on the Bio-Rad CFX96 Real-Time PCR system (Bio-Rad Laboratories Inc., Hercules, CA, USA) in a 20 μL reaction volume containing 50 ng of cDNA template, 10 μmol of primers, 10 μL of 2× SYBR Green real-time PCR master mix, and deionized water. The amplification procedure comprised 40 cycles at 95 °C for 30 s, 95 °C for 5 s, and 60 °C for 30 s. The rice *OsActin1* was used as the internal reference gene for RT-qPCR analysis of gene expression level. All primers for RT-qPCR in this study is listed in Table S1.

### 4.5. Detection of Auxin IAA Distribution by DR5 Promoter-GUS Experiment

The genetic materials of *DR5 promoter-GUS* plants used in this study were previously described by Li et al. [34]. Root tips from 10-day-old plants were examined for GUS-histochemical staining and microscopic observation under Leica DM5000 B microscope (Leica, Wetzlar, Germany) following the protocol by Zhou et al. [68].

## Data Availability

The data presented in this study are available in the article and Supplementary Materials.

## Supplementary Materials

The following supporting information can be downloaded at: https://www.mdpi.com/article/10.3390/plants13040476/s1, Table S1: The primers used for RT-qPCR analysis in this study.
