# Supplementary material for: Characterization of OsPIN2 Mutants Reveal Novel Roles for Reactive Oxygen Species in Modulating Not Only Root Gravitropism but Also Hypoxia Tolerance in Rice Seedlings
Source: Plants (Basel). 2024 Feb 7;13(4):476. doi: 10.3390/plants13040476 (PMC10892736; doi:10.3390/plants13040476)
Supplement: Supplementary file 1 [file plants-13-00476-s001.zip › plants-2816858-supplementary.pdf]

Table S1. The primers used for RT-qPCR analysis in this study.

Gene ID	Primer name	Primer sequences
LOC_Os03g50885	OsActin1-F	GTGGTCGCCCCTCCTGAAAG
	OsActin1-R	GGCTTAGCATTCTTGGGTCCG
LOC_Os01g25820	OsNOX1-F	GGCTTCAATGCCTTCTGGT
	OsNOX1-R	ATGGCTCCTAAACAACCGA
LOC_Os01g53294	OsNOX2-F	ATCCGCAAAATAAGCACCTCT
	OsNOX2-R	CAGTAGCCCATCACATCAAAGAC
LOC_Os01g61880	OsNOX3-F	TCAAGGCAGCGATTTACCC
	OsNOX3-R	CTCGCAAGCCTTCCCAAA
LOC_Os05g38980	OsNOX4-F	CACAAGGTTATCGCACTGACG
	OsNOX4-R	AGCGATGAGTATGTTGGTTGA
LOC_Os05g45210	OsNOX5-F	CCAGTGGGTGGGAAAAGTG
	OsNOX5-R	GTCCGATTGGCGGGTAAA
LOC_Os08g35210	OsNOX6-F	CCTTTCTCCATCACTTCAGCA
	OsNOX6-R	GGGCCATCTACAAGCAACC
LOC_Os09g26660	OsNOX7-F	GTCAAATGCTTATGCTGTCA
	OsNOX7-R	TGTCCAGTCTCCGTTTGTT
LOC_Os11g33120	OsNOX8-F	ACCTTACCTGCGATTTTCCA
	OsNOX8-R	ACGAAGCAGTGGTGGGAGT
LOC_Os12g35610	OsNOX9-F	TACTTCGGGCAGACACGGAT
	OsNOX9-R	GCGGGTTGCTGTCACTAAG
LOC_Os08g09860	OsGOX6-F	ATGCCCGTAATGATTGCTCC
	OsGOX6-R	GGCTCTTCTGACGAACTGCTC
LOC_Os09g11460	OsSUB1B-F	GACGAGGTTCGCCGCATCTA
	OsSUB1B-R	GTCGGAGCAGCACTCGATGA
